# A stabilized HIV-1 envelope glycoprotein trimer fused to CD40 ligand targets and activates dendritic cells

**DOI:** 10.1186/1742-4690-8-48

**Published:** 2011-06-20

**Authors:** Mark Melchers, Katie Matthews, Robert P de Vries, Dirk Eggink, Thijs van Montfort, Ilja Bontjer, Carolien van de Sandt, Kathryn David, Ben Berkhout, John P Moore, Rogier W Sanders

**Affiliations:** 1 Laboratory of Experimental Virology, Department of Medical Microbiology Center for Infection and Immunity Amsterdam (CINIMA), Netherlands; 2 Department of Microbiology and Immunology, Weill Medical College of Cornell University, New York, USA

## Abstract

**Background:**

One reason why subunit protein and DNA vaccines are often less immunogenic than live-attenuated and whole-inactivated virus vaccines is that they lack the co-stimulatory signals provided by various components of the more complex vaccines. The HIV-1 envelope glycoprotein complex (Env) is no exception to this rule. Other factors that limit the induction of neutralizing antibodies against HIV-1 lie in the structure and instability of Env. We have previously stabilized soluble trimeric mimics of Env by introducing a disulfide bond between gp120 and gp41 and adding a trimer stabilizing mutation in gp41 (SOSIP.R6 gp140).

**Results:**

We further stabilized the SOSIP.R6 gp140 using a GCN4-based isoleucine zipper motif, creating SOSIP.R6-IZ gp140. In order to target SOSIP.R6-IZ to immune cells, including dendritic cells, while at the same time activating these cells, we fused SOSIP.R6-IZ to the active domain of CD40 ligand (CD40L), which may serve as a '*cis*-adjuvant'. The Env component of the SOSIP.R6-IZ-CD40L fusion construct bound to CD4 and neutralizing antibodies, while the CD40L moiety interacted with CD40. Furthermore, the chimeric molecule was able to signal efficiently through CD40 and induce maturation of human dendritic cells. Dendritic cells secreted IL-6, IL-10 and IL-12 in response to stimulation by SOSIP.R6-IZ-CD40L and were able to activate naïve T cells.

**Conclusions:**

Chimeric HIV-1 gp140 - CD40L trimers can target and activate dendritic cells. Targeting and activating immune cells using CD40L and other '*cis*-adjuvants' may improve subunit protein vaccine immunogenicity for HIV-1 and other infectious diseases.

## Background

A vaccine against HIV-1 infection remains elusive. Live-attenuated SIV/HIV vaccines have consistently elicited protective immune responses in monkey models, but this approach is generally considered to be unsafe for human use [1]. Despite recent setbacks, recombinant viral vectors such as adenovirus that express HIV-1 proteins continue to be evaluated, but they do not elicit neutralizing antibody (NAb) responses efficiently [2]. Mucosal immunity against HIV-1 has also proven hard to elicit by any vaccine approach, a substantial problem considering that the virus is sexually transmitted [3].

Inducing high titers of broadly active NAbs is a major goal of many HIV-1 vaccine approaches that has not yet been achieved. The most common approaches are based around protein subunit immunogens that mimic the native viral envelope glycoprotein complex (Env), which is the only target for NAbs. Unfortunately, most anti-Env antibodies are unable to neutralize primary HIV-1 isolates. Vaccines based on monomeric gp120 proteins failed to confer protection in efficacy trials [4,5]. The difficulty in inducing NAbs is in part rooted in the structure of the Env complex, which has evolved multiple defenses that limit the induction and binding of such antibodies. Thus, various structural devices shield otherwise vulnerable conserved neutralization epitopes such as the receptor binding sites [6-8], and highly immunogenic but non-neutralizing epitopes exposed on non-functional forms of Env serve as immune decoys [9]. Env sequence variation is another major obstacle for vaccine development that has not been solved [10].

In common with the approaches of other research groups, we have engineered recombinant versions of the native, trimeric HIV-1 Env complex to try to overcome some of these problems. Our approach has been to stabilize the gp120-gp41 (SOS gp140; [11]) and the gp41-gp41 (SOSIP gp140; [12]) interactions, so as to maintain the complex in a trimeric configuration after cleavage of the gp120-gp41 linkage. In general, Env trimers of various designs, including SOSIP gp140s, are superior to gp120 monomers for NAb induction [13-15]. Unfortunately, none of the improvements has yet been sufficient to solve the 'neutralizing antibody problem'.

One general limitation to subunit protein vaccines and DNA plasmid vaccines that encode such proteins is their poor immunogenicity compared to live-attenuated or inactivated viral vaccines. Moreover, the HIV-1 Env proteins are particularly poor immunogens. Thus, the anti-Env titers in vaccinated individuals are relatively low compared to those raised against other protein antigens, and they decay with an unusually short half-life of 30-60 days [16]. It was recently shown that Env proteins predominantly induce short-lived memory B cell-dependent plasma Abs in the settings of HIV-1 envelope vaccination and HIV-1 infection [17]. Other factors such as the magnitude and duration of the antibody response, affinity maturation and the induction of B cell memory are also relevant to the design of an effective B-cell vaccine against HIV-1. The poor performance of Env-based vaccines in these areas is rooted in the structure of the Env complex and its interaction with the immune system. By providing additional stimulatory signals to B cells it seems possible not only to increase the extent and duration of antibody production, but also improve their quality, probably because the increase in B cell stimulation promotes antibody affinity maturation [18]. For example, B cell stimulation through Toll-like receptors (TLRs) improves both the affinity and the neutralizing activity of antibodies against respiratory syncytial virus (RSV) [18].

The addition of co-stimulatory molecules is one way to enhance or tune the immune response to antigens. Covalently linking of adjuvants or co-stimulatory molecules to the antigen appears to be significantly superior to simply administering them as a mixture [19,20]. A few attempts to conjugate HIV-1 Env immunogens to co-stimulatory molecules to improve antibody responses have been made, but with some success [21-23]. Another approach to the problem, using model antigens, showed that antigen targeting to dendritic cells (DC) via lectins such as DC-SIGN, DEC205, DCIR2 or Clec12A can augment antigen-specific immune responses [24-27]. This kind of strategy has not yet been tested using HIV-1 Env.

The intent of this study was to target trimeric HIV-1 Env proteins directly to DC while simultaneously supplying a powerful stimulatory signal to these cells [28]. To do this, we fused the Env proteins to CD40L, a TNF-superfamily member that is normally expressed on T helper cells. By binding to CD40 on DC and B cells, CD40L provides stimulatory signals that are a key element in T cell help. CD40L promotes the antigen-presenting function and migratory capacities of antigen-presenting cells (APCs), enhances the production of pro-inflammatory cytokines such as IL-12 and TNFα, and helps induce memory T cells [29-31]. Furthermore, CD40L activates humoral immunity by promoting the proliferation of B cells, their differentiation to antibody-secreting plasma cells and memory B cells, their selection in germinal centers, and Ig class-switching [31,32]. CD40L and agonistic anti-CD40 antibodies have been used successfully as adjuvants in various immunization models [33-40], as well as in HIV-1 virus-like particle based approaches [41-43]. Finally, its use in humans appears to be safe [44]. Here, we describe the design and construction of a soluble trimeric gp140-CD40L fusion protein that binds CD4, anti-Env NAbs and CD40, induces signaling through CD40 and activates DC *in vitro*.

## Materials and methods

### Plasmid construction

**Figure 1:**
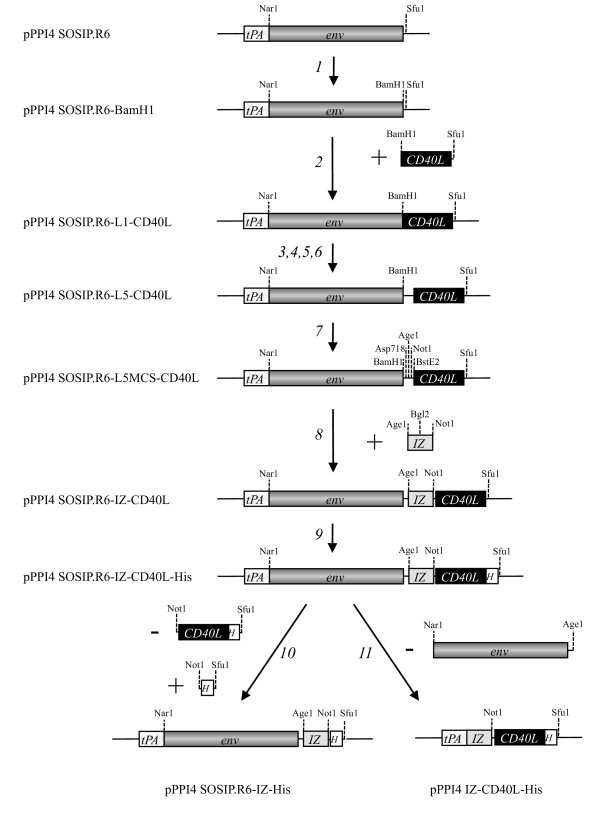
**Cloning strategy**. The following steps were carried out to obtain the constructs used in subsequent experiments: 1. Introduction of a unique BamH1 site at the C-terminus of the *env *gp140 sequences in the pPPI4 SOSIP.R6 plasmid; 2. Insertion of the sequences encoding mouse CD40L amino acids 118-261, amplified from J558 cells and cloned using BamH1 and Sfu1; 3,4,5,6. Insertion of various linkers between *env *and CD40L sequences, generated by PCR and cloned using BamH1 and Sfu1; 7. Introduction of the unique restriction sites for Asp718, Age1, Not and BstE2 in the linker (L5) between *env *and CD40L; 8. Insertion of the sequences encoding an isoleucine zipper (IZ) based trimerization domain, generated by annealing of oligonucleotides and cloned using Age1 and Not1; 9. Insertion of sequences encoding an oligohistidine tag at the C-terminus of CD40L; 10. Deletion of the sequences encoding CD40L to generate a plasmid encoding SOSIP.R6 gp140 fused to the IZ domain followed by a oligohistidine tag; 11. Deletion of the *env *sequences, generating a construct encoding a trimeric CD40L control molecule. The non codon-optimized mouse CD40L sequences were replaced by codon-optimized sequences for mouse and human CD40L using Not1 and Sfu1 (not shown in the figure). All constructs were verified by sequencing. The codon-optimized constructs were used in all following experiments, mouse or human depending on the application. More details are provided in the Materials and Methods section.

The pPPI4 plasmid (Progenics Pharmaceuticals Inc., Tarrytown, NY) containing a codon-optimized stabilized gp140 gene that is based on of the subtype B, R5 isolate JR-FL has been described elsewhere (SOSIP.R6 gp140; [11,12,45]. To facilitate subsequent cloning steps, we first introduced a BamH1 site at the C-terminus of SOSIP.R6 gp140. This modification changed the most C-terminal amino acid of the natural gp140 protein (Y681I), and added one more amino acid (682L). These changes did not adversely affect the folding and secretion of SOSIP.R6 gp140 proteins (data not shown). The cloning steps are indicated in Figure 1.

The gene plasmids encoding the functional domain (amino acids 118 to 261) of murine CD40L was amplified from the mouse fibroblast cell line J558 (American Type Culture Collection, Rockville, MD), using the Expand PCR system according to the manufacturer's instructions (Roche, Mannheim, Germany). The PCR was performed with sense and antisense primers (5'mCD40L1BamH1 [5'-CTCATACTCATAGGATCCTCGATCCTCAAATTGCAGC-3'] and 3'mCD40LSfu1 [5'-CTCATACTCATATTCGAATTAGAGTTTGAGTAAGCC-3']). The PCR product was cloned downstream of the SOSIP.R6 ORF in pPPI4-SOSIP.R6 using BamHI and SfuI, creating pPPI4-SOSIP.R6-L1-CD40L. The plasmids pPPI4-SOSIP.R6-L2-CD40L and pPPI4-SOSIP.R6-L3-CD40L were created by PCR amplification using pPPI4-SOSIP.R6-L1-CD40L as the template and the following 5' primers and, in both cases, 3'mCD40LSfu1: 5'mCD40L2BamH1: [5'-CTCATACTCATAGGATCCTCGGTGGAGGTAGCGATCCTCAA ATTGCAGC-3']; 5'mCD40L3BamH1: [5'-CTCATACTCATAGGATCCTCGGTGGAGGTAGCGGTGGAGG TGATCCTCAAATTGCAGC-3']. The resulting BamH1-Sfu1 fragments containing the linker sequences and amino acids 118-261 from CD40L were then cloned behind the SOSIP.R6 gp140 sequences.

The pPPI4-SOSIP.R6-L4-CD40L plasmid was generated by PCR amplification, with pPPI4-SOSIP.R6-L3-CD40L as the template and primers 5'mCD40L4BamH1: [5'-CTCATACT CATAGGATCCTCGGCGGTGGCGGTAGCGGTGGTGGAGGTAGC-3'] and 3'mCD40LSfu1. Plasmid pPPI4-SOSIP.R6-L5-CD40L was generated by PCR amplification using pPPI4-SOSIP.R6-L4-CD40L as a template and primers 5'mCD40L5BamH1: [5'-CTCATACTCATAGG ATCCTCGGTGGAGGTGGAAGCGGCGGTGGCGGT-3'] and 3'mCD40LSfu1. These steps created the following spacers between SOSIP.R6 and mCD40L: L1: No spacer; L2: GGGS; L3: GGGSGGG; L4: GGGGSGGGGSGGG; L5 GGGGSGGGGSGGGGSGGG.

To facilitate subsequent cloning steps, the linker region of pPPI4-SOSIP.R6-L5-CD40L between Env and CD40L was further modified to introduce the restriction sites for Asp718, Age1, Not1 and BstE2 (pPPI4-SOSIP.R6-L5MCS-CD40L), creating the 18 amino acid linker sequence GGGGTGGGGTGGGGRGGG (non-silent changes are underlined). The resulting sequence modifications did not adversely affect the secretion of the SOSIP.R6-L5-CD40L fusion protein (data not shown).

A DNA fragment encoding a codon-optimized isoleucine zipper motif (IZ) based on GCN4 (AGAATGAAGCAGATCGAGGACAAGATCGAGGAGATCCTGAGCAAGATCTACCACA TCGAGAACGAGATCGCCAGAATCAAGAAGCTGATCGGCGAGAGA, which encodes the peptide sequence RMKQIEDKIEEILSKIYHIENEIARIKKLIGER [46]), was annealed using two 5'-sense oligonucleotides, 5'IZ1Age1Bgl2: 5'CCGGTAGAATGAAGCAGATCGAGGA CAAGATCGAGGAGATCCTGAGCAA-3' and 5'IZ2Bgl2Not1: 5'-GATCTACCACATCGAGAAC GAGATCGCCAGAATCAAGAAGCTGATCGGCGAGAGAGGC-3' and the two antisense oligonucleotides 3'IZ1Age1Bgl2: 5'-GATCTTGCTCAGGATCTCCTCGATCTTGTCCTCGATCT GCTTCATTCTA-3' and 3'IZ2Bgl2Not1: 5'-GGCCGCCTCTCTCGCCGATCAGCTTCTTGATTC TGGCGATCTCGTTCTCGATGTGGTA-3', leading to a double stranded DNA fragment with a 5'AgeI site (single underline), a Bgl2 site (double underlined) and a 3' NotI site (dotted underline). This fragment was cloned into pPPI4-SOSIP.R6-L5MCS-CD40L using AgeI and NotI, leaving a linker of 11 amino acids (GGGGTGGGGTG) between the SOSIP.R6 gp140 and IZ moieties, and a 6-amino acid linker (GGRGGG) between IZ and CD40L. Finally, we added a C-terminal oligo-Histidine tag (HHHHHHHHH) using the Quickchange mutagenesis kit (Stratagene, La Jolla, CA).

We also created a similar plasmid without the sequences encoding CD40L (pPPI4-SOSIP.R6-IZ), by replacing the NotI-SfuI fragment (CD40L) by one containing only the oligo-Histidine tag [47]. Codon-optimized genes encoding the extracellular domain of the human and mouse versions of CD40L (amino acids 120 to 261) were synthesized (Mr. gene, Regensburg, Germany) and cloned behind SOSIP.R6-IZ using Not1 and Sfu1. The pPPI4-IZ-CD40L plasmid encoding trimeric CD40L without gp140 was constructed by cutting out the Env-encoding sequences from pPPI4-SOSIP.R6-IZ-hCD40L using Nar1 and Age1, followed by Klenow blunting and self-ligation.

The sequence integrity of all clones was confirmed prior to use. The amino acid numbering of SOSIP.R6 gp140 is based on HXB2 Env.

### Cell culture and transient transfection

293T cells were transiently transfected with Env using linear polyethylenimine as described previously [48]. Briefly, Env-encoding plasmids (or plasmid DNA for mock transfections) were diluted to 0.1 × the culture volume and mixed with Dulbecco's Modified Eagle's Medium (Invitrogen, Breda, The Netherlands). A volume of 0.15 × the culture volume of a 1 mg/ml solution of linear Polyethylenimine (PEI, MW 25,000, Polysciences Europe GmbH, Eppenheim, Germany) was then added and mixed. After incubation for 20 min, the DNA-PEI mix was added to the cells for 4 h before replacement with the same culture medium supplemented with 10% fetal bovine serum (FBS) (HyClone, Perbio, Etten-Leur, The Netherlands), penicillin, streptomycin, and MEM non-essential amino acids (0.1 mM, Invitrogen). Env-containing supernatants were harvested 48 h after transfection. All supernatants used for functional assays were concentrated 60x.

### Concentrating the proteins

Cell supernatants from transient transfections were concentrated using Amicon Ultra-15 Centrifugal Filter Units with 100 kD MWCO filter (Millipore, Amsterdam Zuidoost, The Netherlands), except for IZ-CD40L for which a 30 kD MWCO filter was used due to its lower molecular weight. The concentration was performed according to the manufacturer's instructions.

### SDS-PAGE, Blue Native PAGE and Western blotting

SDS-polyacrylamide gel electrophoresis (SDS-PAGE) and Western blotting were performed according to established protocols using the anti-gp120 V3 loop MAb PA-1 (1:20,000; final concentration, 50 ng/ml; Progenics) [49] and horseradish peroxidase-labeled goat-anti-mouse IgG (1:5,000, Jackson Immunoresearch, Suffolk, UK). Luminometric detection of envelope glycoproteins was performed using the western lightning ECL system (PerkinElmer, Groningen, The Netherlands). Blue Native (BN)-PAGE was carried out with minor modifications to the published method [12,50,51]. Thus, purified protein samples or cell culture supernatants were diluted with an equal volume of a buffer containing 100 mM 4-(N-morpholino) propane sulfonic acid (MOPS), 100 mM Tris-HCl, pH 7.7, 40% glycerol, 0.1% Coomassie blue, just prior to loading onto a 4 to 12% Bis-Tris NuPAGE gel (Invitrogen). Typically, gel electrophoresis was performed for 3 hrs at 125 V using 50 mM MOPS, 50 mM Tris, pH 7.7 as running buffer.

### Gel filtration analysis

Concentrated culture supernatants, derived from transiently transfected 293T cells were fractionated on a Superose-6 column in PBS using an AKTA FPLC, (GE Healthcare Lifesciences, Diegem, Belgium), followed by analysis by standard SDS-PAGE and western blot using MAb PA-1 (Progenics).

### Immunoprecipitation assays

Supernatants were concentrated 25-fold from 293T cells transiently transfected with the SOSIP.R6-IZ-CD40L construct and incubated overnight at 4°C with MAbs, CD4-IgG2 or mouse CD40-Fc in a 500 μl volume containing 100 μl of a 5-fold concentrated RIPA buffer (250 mM Tris-HCl, pH 7.4, 750 mM NaCl, 5% NP-40, 12.5 mM Na-deoxycholate, Complete Protease Inhibitor Cocktail (Roche, Mannheim, Germany)). Next, 50 μl of protein G-coated agarose beads (Pierce Inc./Thermo Fisher Scientific, Etten-Leur, The Netherlands) was added and rotation-mixed for 2 hrs at 4°C. The beads were washed extensively with ice-cold 1x RIPA buffer containing 0.01% Tween 20. Proteins were eluted by heating the beads at 100°C for 5 min in 50 μl of SDS-PAGE loading buffer supplemented with 100 mM dithiothreitol (DTT). The immunoprecipitated proteins were fractionated on 8% SDS-PAGE gels (Invitrogen) at 125 V for 2 h. For exact reagents used see "reagents" section.

### CD40 reporter assays

CD40-293-SEAP cells were used that stably express CD40. In addition, they are stably transfected with the pNiFty2 plasmid (Invivogen, San Diego, CA, USA), which contains the secreted embryonic alkaline phosphatase (SEAP) gene under the control of the ELAM-1 promoter containing five NF-kB binding sites. Cells were seeded in 96-well plates (2 × 10^4 ^cells per well) in Optimem (Invitrogen, Breda, The Netherlands). Concentrated supernatant containing the various Env-CD40L variants were serially diluted in Optimem and added to the cells. The cells were stimulated for 24 hours at 37°C/5% CO2. The same dilution of mock-transfected supernatant served as a negative control. The positive control was a similar dilution of IZ-CD40L containing concentrated supernatant. The production of secreted embryonic alkaline phosphatase (SEAP) was measured according to the manufacturer's protocol (Quanti-blue, InvivoGen). In short, 5 μl of cell-free culture supernatant was transferred after 24 h to a new 96-well plate, mixed with 200 μl Quanti-Blue (QB) (37°C) and incubated for 18 h at 37°C in the dark. Colorimetric detection of SEAP activity was performed by measuring the optical density at 630 nm using a model 550 reader (Bio-Rad, Veenendaal, The Netherlands).

### DC propagation

Peripheral blood mononuclear cells (PBMC) were isolated from buffy coats (New York Blood Center) by Ficoll density gradient centrifugation. Monocytes were isolated from PBMC by positive magnetic cell selection with CD14 microbeads (Miltenyi Biotech, Auburn, CA, USA) according to the manufacturer's recommendations. The sorted monocytes were > 98% pure with contaminating T-cell populations representing < 1% of the cells. Monocytes were subsequently resuspended at 1 × 10^6 ^cells/ml in RPMI 1640 (Cellgro, Manassas, VA, USA) containing 5% human AB serum (Sigma-Aldrich, St. Louis, MO, USA), 100 U/ml penicillin and 100 μg/ml Streptomycin (Hyclone), 2 mM L-glutamine, 1 mM sodium pyruvate, 0.1 mM non-essential amino acids, 25 mM HEPES (Gibco/Invitrogen), plus 1,000 U/ml GM-CSF (Leukine, Sargramostim) and 1,000 U/ml of recombinant human IL-4 (R & D Systems). The monocytes were seeded into 6-well plates (3 × 10^6 ^cells/well) in a final volume of 3 ml, and cultured at 37°C in an atmosphere containing 5% CO_2_. Immature DCs were fed every 2 days with 300 μl of fresh media containing 3,000 U of both GM-CSF and IL-4 to maintain the concentration of cytokines at 1,000 U/ml. The phenotype of the immature DC (iDC) was evaluated on day 5 or 6, when > 90% of cells were CD11c^+ ^HLA-DR^+ ^CD206^++ ^CD209^++ ^and CD14^- ^CD80^- ^CD83^-^.

### Dendritic cell stimulation

The iDCs were gently removed from the 6-well plates, centrifuged at 300 g for 5 min at room temperature and resuspended at 1 × 10^6 ^cells/ml in residual tissue culture supernatant. A total of 5 × 10^5 ^iDC were seeded into 48-well plates in a final volume of 1 ml, and then exposed to the following stimuli for 48 h: 300 μl of 25-fold concentrated supernatants from transfected 293T cells containing approximately 3 μg/ml of SOSIP.R6-IZ or SOSIP.R6-IZ-CD40L protein (mock transfected supernatants were included in all experiments); 3 μg/ml of purified recombinant JR-FL gp120; 10 ng/ml TNF-α and 10 ng/ml IL-1β (R & D Systems); 100 ng/ml LPS derived from Salmonella typhimurium (Sigma-Aldrich); or combinations thereof. An aliquot (10^5^) of the cells was left unstimulated in every experiment, to ascertain the baseline levels of phenotypic markers and cytokine production.

### Immunophenotypic analysis of DC

Before and after in vitro stimulation, the DCs were immunophenotyped using fluorochrome-labeled MAbs to CD11c (clone B-ly6), CD14 (clone mϕp9), CD40 (clone 5C3), CD80 (clone L307.4), CD83 (clone HB15e), CD86 (clone IT2.2), CD206 (clone 19.2), CD209 (clone DCN46) and HLA-DR (clone L243) (BD Biosciences, San Jose, CA, USA). The iDC were transferred into 96-well U-bottomed plates prior to staining, and washed in ice-cold FACS buffer (PBS containing 5% human AB serum), with centrifugation at 300 g and 4°C. 10^5 ^DCs were stained for each MAb combination. Non-specific binding of MAbs to cell surface FcRs was prevented by blocking these receptors with 10% human AB serum for 30 min on ice. MAb cocktails (50 μl) containing pre-titrated antibodies were added to DCs for an additional 30 min on ice. Isotype-matched control MAbs were included in every assay. After staining, the DCs were washed twice and fixed in 1% paraformaldehyde. Four-color analysis was performed using an LSR II flow cytometer and the data were analyzed with FlowJo software (Version 7.2, TreeStar Inc. Ashland, OR). DCs were identified by high forward scatter and side scatter and by their uniform expression of CD11c. Signals from at least 50,000 DC were acquired from each sample.

Cell-free supernatants from DC cultures were collected post-stimulation and immediately frozen at -80°C until analysis. Their contents of IL-6, IL-10, IL-12p70 and TNF-α were determined using commercially available ELISA kits (BD Pharmingen, San Jose, CA, USA) according to the manufacturer's instructions. Absorbance was measured using an Emax precision microplate reader (Molecular Devices, Sunnyvale, CA, USA). The assay detection sensitivity was 2 pg/ml for IL-6 and 4 pg/ml for IL-10, IL-12p70 and TNF-α.

### Activation of naïve CD4^+ ^T-cells

Allogeneic naïve CD4^+ ^T-cells were isolated from frozen PBMC derived from a healthy donor (New York Blood Center) by negative selection using the naïve CD4^+ ^T cell isolation kit II (Miltenyi Biotech). The purity of isolated naïve CD4^+ ^T-cells was assessed following surface staining with the following MAbs (BD Pharmingen): CD3-APC (clone HIT3a), CD4-PERCP (clone SK3), CD45RO-PE (UCHL1) and CD27-FITC (M-T271). Naïve CD4^+ ^T-cells were defined as CD3^+ ^CD4^+ ^CD45RO^- ^CD27^+ ^and purities exceeded 98%. Naïve CD4^+ ^T-cells were co-cultured at a ratio of 1 DC: 10 CD4^+ ^T-cells (10^5 ^DC + 10^6 ^T-cells) in 1 ml final volume in a 48 well plate in the absence or presence of differentially stimulated DC. On day 5, CD4^+ ^T-cells were stained with anti-CD3-APC/CD4-PERCP (as above) and HLA-DR-FITC (clone L243). For assessment of intracellular cytokine expression, CD4+ T-cells were re-stimulated with PMA (100 ng/ml) and ionomycin (1 μg/ml) for 6 h in the presence of brefeldin A (1 μg/ml) for the last 4 h. CD4^+ ^T-cells were stained with anti-CD3, anti-CD4 and anti-CD45RO (as above) and anti-HLA-DR-FITC (clone L243). Intracellular cytokine staining was performed after permeabilization using BD Biosciences Cytofix/Cytoperm solution according to the manufacturer's instructions, followed by incubation at room temperature with anti-IFN-γ-PE (clone B27), anti-IL-4-PE (clone 8D4-8) or isotype matched control (murine IgG1-PE; clone X40). IL-2, IL-4 and IFN-γ levels in the supernatant of DC-T cell co-cultures were measured using a commercially available ELISA kit (BD Pharmingen).

### Reagents

The recombinant human CD40/TNFRSF5/Fc chimera and anti-mouse CD40L monoclonal antibody (cross-reactive with human) were purchased from R&D Systems. MAb 2F5 was obtained from Hermann Katinger through the NIH AIDS Research and Reference Reagent Program (ARRRRP); HIVIg was obtained through the ARRRP from NABI and NHLBI. MAb b12 was donated by Dennis Burton (The Scripps Research Institute, La Jolla, CA, USA); CD4-IgG2, PA-1 and recombinant JR-FL gp120 (expressed in Chinese hamster ovary cells, endotoxin content < 3 EU/ml) were a gift from William Olson (Progenics Pharmaceuticals Inc., Tarrytown, NY, USA).

## Results

### Enhancing SOSIP.R6 gp140 trimer formation

**Figure 2:**
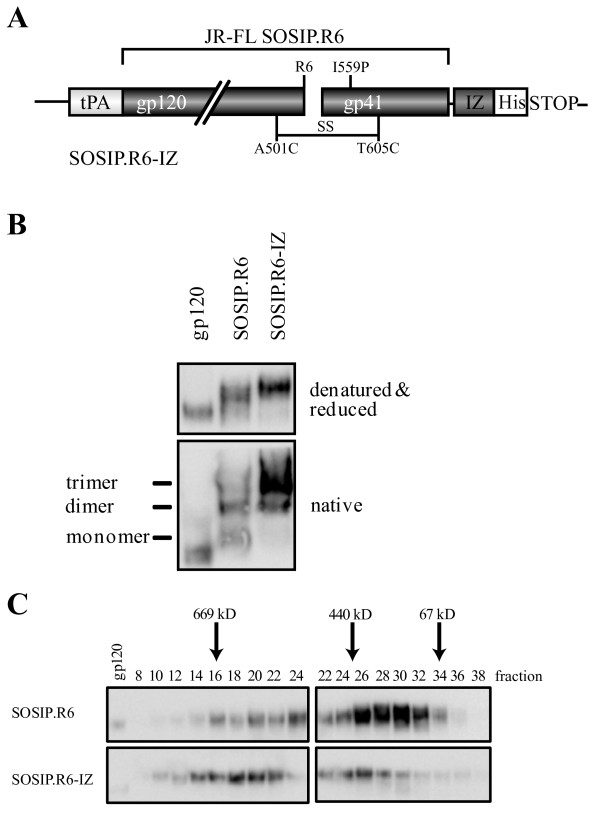
**Improved trimerization of JRFL-SOSIP.R6 gp140 by addition of an heterologous trimerization domain**. **A**. Schematic of the SOSIP.R6.IZ design. The clade B JR-FL gp140 (amino acids 31-681) contains several modifications that have been previously described (see Materials and Methods). Trimer formation was further enhanced by insertion of a GCN4-based isoleucine zipper (IZ) to the C-terminus of SOSIP.R6. **B**. Reducing SDS-PAGE and Blue Native-PAGE analysis of SOSIP.R6 and SOSIP.R6-IZ proteins secreted from transiently transfected 293T cells. Note that no exogenous furin was added in these experiments, therefore the proteins are predominantly (> 90%) uncleaved. **C**. Gel filtration analysis of SOSIP.R6 and SOSIP.R6-IZ proteins. Concentrated culture supernatants, derived from transiently transfected 293T cells, containing the SOSIP.R6 or SOSIP.R6-IZ proteins were fractionated on a Superose-6 column, followed by analysis by SDS-PAGE and western blot. The elution of standard proteins is indicated.

We have previously described modifications that improve the stability of soluble, cleaved gp140 trimers, based on the R5 subtype B isolate JR-FL [11]. The amino-acid sequence of gp120 and the gp41 ectodomain was modified as follows (Figure 2A). We introduced: (i) a disulfide bond between residues 501 in gp120 and 605 in gp41 (A501C, T605C; [11]); (ii) a trimer-stabilizing substitution in gp41 (I559P; [12]); (iii) a sequence-enhanced site for furin cleavage (RRRRRR; [45]). Despite these modifications, the resulting JR-FL SOSIP.R6 gp140 protein (hereafter called SOSIP.R6) is expressed as heterogeneous oligomers, with monomers, dimers and tetramers present as well as the desired trimers (Figure 2B).

In previous studies, the addition of heterologous trimerization motifs has been shown to improve gp140 trimer formation [52]. We therefore introduced a GCN4-based isoleucine zipper (IZ) sequence [46] at the C-terminus of SOSIP.R6 (Figure 2A). In addition, we added an octahistidine (His) motif immediately C-terminal to the IZ trimerization domain, with flexible 11 and 6 amino acid linkers placed between SOSIP.R6-IZ and IZ-His tag, respectively (Figure 2A). The optimal linker length was determined in concurrent studies (see below). The resulting SOSIP.R6-IZ and unmodified SOSIP.R6 proteins were expressed transiently in 293T cells and then analyzed by SDS-PAGE and Blue Native (BN)-PAGE. Both SOSIP.R6 proteins were efficiently expressed (Figure 2B, top panel). As expected, the unmodified SOSIP.R6 was secreted as a mixture in which monomers, dimers and trimers were present. The proportion of trimers was markedly greater, however, for the SOSIP.R6-IZ protein (~90%), presumably because of the impact of the heterologous trimerization motif (Figure 2B, bottom panel).

We, next, studied the SOSIP.R6 and SOSIP.R6-IZ proteins using analytical size exclusion chromatography on a Superose-6 column, compared to standard proteins of defined molecular weight (Figure 2C). Analysis of the eluted Env-protein components by SDS-PAGE and Western blotting confirmed that multiple oligomeric gp140 forms were present [12]. We previously reported that SOSIP.R6 gp140 monomers, dimers and trimers were eluted from a Superdex-200 size exclusion column at positions corresponding to apparent molecular weights of 240, 410 and 520 kDa, respectively. Here, using Superose-6 columns that allow greater resolution at the higher end of the molecular weight range of interest, we observed that most of the SOSIP.R6 protein forms were eluted in volumes corresponding to apparent molecular weights in the range 150-550 kDa, which is consistent with the presence of monomers, dimers and trimers. In contrast, the SOSIP.R6-IZ protein forms were more homogeneous, with a predominant elution peak of ~600 kDa that is consistent with the enrichment of trimers. Hence, the gel filtration analysis confirms the SDS-PAGE and BN-PAGE studies and shows that the addition of the IZ motif enhances SOSIP.R6 trimer formation and/or stability.

When SOSIP.R6 proteins are expressed in 293T cells they are incompletely cleaved at the juncture between gp120 and the gp41 ectodomain, but the efficiency of cleavage is increased to ~95% by the co-transfection of a plasmid expressing furin [11,45]. In contrast, even in the presence of exogenous furin, the SOSIP.R6-IZ proteins were only partially cleaved (< 50% processing; data not shown), and processing was minimal (< 10%) when furin was not co-transfected (Figure 2B). The addition of sequence motifs to the C-terminus of the gp41 ectodomain appears to interfere with cleavage at a site several hundred residues upstream. We are now studying the underlying reasons to try to find a solution to this problem because uncleaved Env is antigenically different from cleaved Env [11,15,53,54]. In the absence of a solution to the cleavage problem, we elected to not co-transfect furin when expressing the various Env proteins outlined below, which are therefore all predominantly uncleaved.

### Construction of a trimeric SOSIP.R6-IZ-CD40L fusion protein

**Figure 3:**
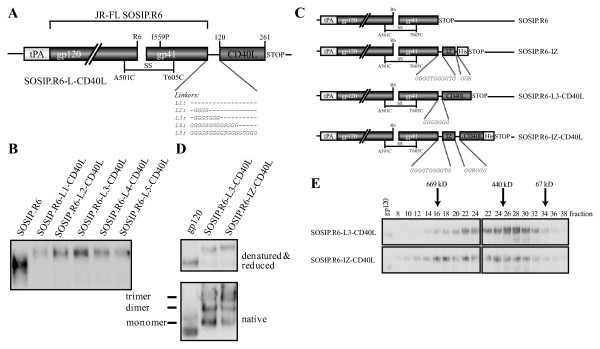
**JRFL-SOSIP.R6-IZ-CD40L design and construction**. **A**. Schematic of the SOSIP.R6.L-CD40L design and its various linkers. **B**. Optimization of the linker between SOSIP.R6 and CD40L using reducing SDS-PAGE analysis of transiently expressed SOSIP.R6-L-CD40L with the different linkers. **C**. Schematic of the constructs mainly used in this study. **D**. Reducing SDS-PAGE and Blue Native-PAGE analysis of SOSIP.R6-L3-CD40L and SOSIP.R6-IZ-CD40L proteins secreted from transiently transfected 293T cells. **E**. Gel filtration analysis of SOSIP.R6-L3-CD40L and SOSIP.R6-IZ-CD40LHis proteins. Concentrated culture supernatants, derived from transiently transfected 293T cells, containing the SOSIP.R6-L3-CD40L or SOSIP.R6-IZ-CD40LHis proteins were fractionated on a Superose-6 column, followed by analysis by SDS-PAGE and western blot. The elution of standard proteins is indicated.

We hypothesized that we could increase the immunogenicity of Env trimers by targeting the protein to DC and at the same time providing a strong activation signal to these DCs. We, therefore, fused the extracellular domain of human codon-optimized CD40L, consisting of amino acids 120 to 261 and including the CD40 binding site, to the C-terminus of SOSIP.R6 (Figure 3A). To allow the SOSIP.R6 and the CD40L components to fold independently and the fusion protein to be secreted efficiently, we added flexible glycine-rich linkers between the two elements. Since the optimal linker length could only be established empirically, we compared linkers of 0, 4, 7, 13 and 18 residues (constructs L1-L5; Figure 3A). The different SOSIP.R6-L-CD40L fusion proteins were expressed transiently in 293T cells and the supernatants analyzed by SDS-PAGE and western blotting (Figure 3B). Linkers L2-L4 (4-13 residues) allowed the most efficient secretion of SOSIP.R6-CD40L; having no linker or a longer linker resulted in lower expression levels (Figure 3B). Based on these results, and also cloning considerations, subsequent constructs contained an 11-residue linker between the gp140 and C-terminal components.

CD40L needs to be trimeric to be active. When soluble CD40L is expressed, it is however mostly in the monomeric and therefore inactive state [55-57]. Since the IZ trimerization domain enhanced the trimerization of SOSIP.R6 gp140, an IZ motif was inserted between SOSIP.R6 and CD40L (Figure 3C). SDS-PAGE and BN-PAGE analyses showed that SOSIP.R6-IZ-CD40L was secreted efficiently from transiently transfected 293T cells and predominantly in the trimeric form (Figure 3D).

Analytical size exclusion chromatography confirmed these promising results (Figure 3E). The SOSIP.R6-L3-CD40L protein was eluted in volumes corresponding to molecular weights between 150 and 550 kDa, which is consistent with it being mostly monomers, dimers and trimers. The elution profile was similar to that of unmodified SOSIP.R6 (Figure 2C), but with a small shift to higher sizes caused by the presence of the CD40L moiety. The peak elution volume of the chimeric SOSIP.R6-IZ-CD40L protein was consistent with it being a trimer of ~600 kDa (Figure 3E), confirming that the IZ motif enhanced trimerization. Compared to what was expected from the BN-PAGE analysis, a significant proportion of the proteins eluted from the gel filtration column were monomers and dimers, possibly because some trimers dissociate during elution from the columns.

Similar to the parental SOSIP.R6-IZ construct, the SOSIP.R6-IZ-CD40L fusion construct was not cleaved at the gp120-gp41 junction, which may affect its antigenic structure and perhaps its ability to induce NAbs. However, since our immediate goal was to investigate co-stimulation by the CD40L component, we continued to use the uncleaved fusion construct.

### SOSIP.R6-IZ-CD40L binds to CD4, CD40 and neutralizing antibodies

**Figure 4:**
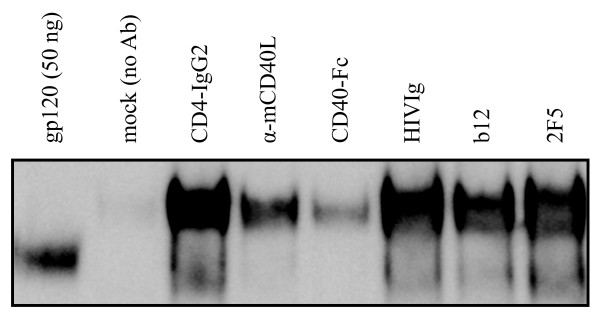
**Recognition of SOSIP.R6-IZ-CD40L by antibodies, CD4 and CD40**. The SOSIP.R6-IZ-CD40L protein was immunoprecipitated by CD4-IgG2, CD40-Fc, HIVIg, and by antibodies to CD40L, gp120 (b12) or gp41 (2F5), followed by reducing SDS-PAGE and western blot analysis. The left lane contains 50 ng JR-FL gp120 as a loading control. The second sample contains an immunoprecipiation reaction without primary Ab. The lower band visible in lanes 3-7 represents gp120 from residual (< 10%) cleaved protein.

To investigate whether the SOSIP.R6 and CD40L components of the chimeric construct were properly folded and functional, we measured the binding to specific ligands. The SOSIP.R6-IZ-CD40L protein was immunoprecipitated efficiently from concentrated supernatant by pooled Ig from HIV-infected individuals (HIVIg) and by NAbs against several gp120 or gp41 epitopes, specifically b12 to the CD4 binding site, 17b to a CD4-induced epitope and 2F5 to the MPER region (Figure 4 and data not shown). Furthermore, the fusion protein bound to the viral receptors CD4 (Figure 4) and DC-SIGN (data not shown). We next performed immunoprecipitations with a neutralizing antibody to CD40L and a CD40-Fc construct (Figure 4). The antibody recognized the CD40L domain of the fusion protein, which was also able to interact with CD40. Thus, the chimeric SOSIP.R6-IZ-CD40L molecule is capable of interacting with relevant receptors and NAbs.

### SOSIP.R6-IZ-CD40L activates NF-κB through CD40

**Figure 5:**
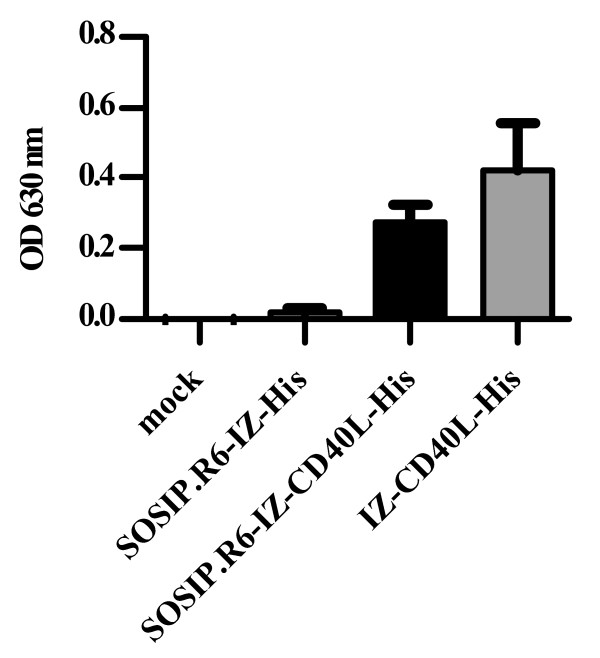
**SOSIP.R6-IZ-CD40L activates NF-κB through CD40**. 293T-CD40 cells were incubated for 18h with mock supernatant or supernatant containing SOSIP.R6-IZ, SOSIP.R6-IZ-CD40L or IZ-CD40L after which SEAP activity in the supernatant was measured. Bars indicated are mean of two experiments + SEM.

To determine whether SOSIP.R6-IZ-CD40L was biologically active, we used a HEK 293-derived CD40 reporter cell line that overexpresses CD40 and produces secreted embryonic alkaline phosphatase (SEAP) when CD40 ligation activates NF-κB [58]. We therefore transiently expressed SOSIP.R6-IZ, trimeric CD40L without SOSIP.R6 (IZ-CD40L) and SOSIP.R6-IZ-CD40L in 293T cells with mock transfected supernatant serving as a negative control. The positive control, concentrated supernatant containing IZ-CD40L proteins, activated NF-κB, as measured by SEAP release (Figure 5A). The concentrated supernatant containing SOSIP.R6-IZ-CD40L fusion protein, but not SOSIP.R6-IZ and mock supernatant, also induced SEAP activity, indicating that the CD40L component was capable of CD40 ligation and signaling through CD40L consistent with the protein being trimeric (Figure 5A).

### SOSIP.R6-IZ-CD40L induces DC maturation

**Figure 6:**
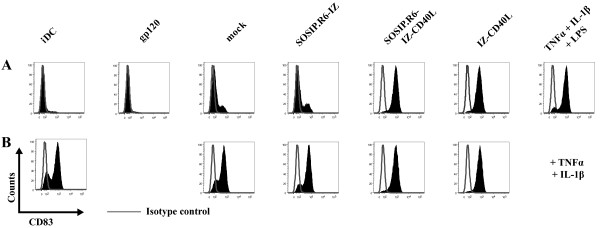
**SOSIP.R6-IZ-CD40L induces DC maturation**. **A**. Monocyte-derived iDC were cultured for 48h in the presence of SOSIP.R6-IZ, SOSIP.R6-IZ-CD40L or control stimuli. The expression of the maturation marker CD83 was monitored by FACS. **B**. CD83 was measured on DC stimulated for 48 h with a combination of TNF-α/IL-1β and SOSIP.R6-IZ, SOSIP.R6-IZ-CD40L or control stimuli. Gray lines represent the isotype-matched control. Cells were double-stained for CD11c and CD83 and CD83 Histograms of cells gated on CD11c are shown (> 25,000 events).

CD40L is an important co-stimulatory molecule for DC during DC-T cell interactions. We therefore investigated whether SOSIP.R6-IZ-CD40L was able to activate DC, using expression of CD83, a well-characterized DC maturation marker, as an endpoint. iDC were treated for 48 h with concentrated 293T supernatant containing SOSIP.R6-IZ-CD40L and, for comparison, with a standard maturation cocktail (TNF-α/IL-1β/LPS, positive control) or concentrated supernatant containing IZ-CD40L, SOSIP.R6-IZ or monomeric gp120. CD83 expression on unstimulated iDC served as a baseline (Figure 6A). An additional DC culture was exposed to supernatants from mock-transfected 293T cells to control for the presence of factors released from these cells (Figure 6A).

Purified, monomeric gp120 did not induce DC maturation (4.9% CD83^+ ^cells, compared to 7.0% on untreated iDC), as reported previously (Figure 6A) [59]. A low level of CD83 up-regulation (19.8% CD83^+^) occurred when DC were treated with supernatant from mock-transfected 293T cells, which is probably attributable to contaminant cytokines or other immunomodulatory proteins. CD83 expression was similar (24.9% CD83^+^) on cells treated with the SOSIP.R6-IZ negative control protein. In contrast, exposure of DC to IZ-CD40L or SOSIP.R6-IZ-CD40L caused almost all the cells to upregulate CD83 (94.8% and 95.5% CD83^+ ^respectively), an outcome comparable to treatment with the TNF-α/IL-1β/LPS maturation cocktail (89.5% CD83^+^). Similar results were obtained using CD80 expression as an alternative marker for DC maturation, while a converse trend was apparent for expression of DC-SIGN (CD209) and the mannose receptor (CD206), two cell surface proteins that are down-regulated when DC mature (data not shown).

We also assessed whether SOSIP.R6-IZ-CD40L and the various control proteins could augment DC maturation induced by TNF-α/IL-1β (Figure 6B). Stimulation by TNF-α/IL-1β induced CD83 expression on 75.6% of the DC (Figure 6B). Adding the mock or SOSIP.R6-IZ supernatants to the TNF-α/IL-1β cocktail had a marginal effect (80.0% and 84.8% CD83^+^, respectively). However, combining SOSIP.R6-IZ-CD40L or IZ-CD40L with TNF-α/IL-1β increased the number of CD83^+ ^cells to 94.8% and 93.6%, respectively. Thus, SOSIP.R6-IZ-CD40L is able to activate DC to at least the same extent as a trimeric CD40L protein.

### SOSIP.R6-IZ-CD40L induces secretion of IL-6, IL-10, IL-12 and TNF-α

**Figure 7:**
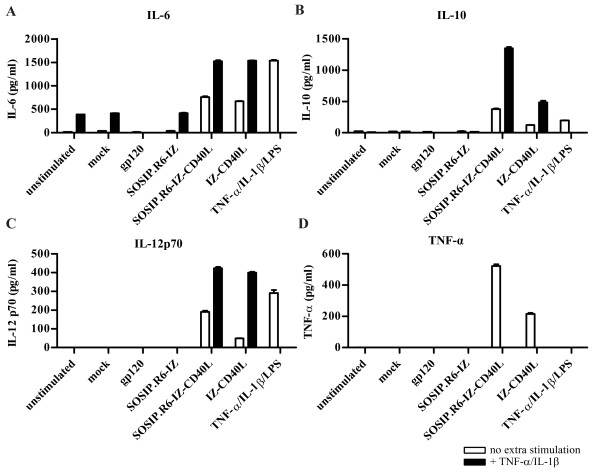
**SOSIP.R6-IZ-CD40L induces cytokine secretion by DC**. IL-6 **(A)**, IL-10 **(B)**, IL-12 **(C) **and TNF-α **(D) **levels in the supernatant of DC stimulated for 48 h with SOSIP.R6-IZ, SOSIP.R6-IZ-CD40L or control stimuli (white bars), or a combination of TNF-α/IL-1β and gp140-IZ, gp140-IZ-CD40L or control stimuli (black bars) were measured by ELISA. Data are representative for three independent experiments. Bars indicated are mean + SD.

Since the particular combination of cytokines secreted by activated DC is central in defining the subsequent immune responses, we wished to identify whether SOSIP.R6-IZ-CD40L-treated cells released IL-6, IL-10, IL-12 and TNF-α. Exposure of DC to gp120, mock supernatant or SOSIP.R6-IZ did not trigger the secretion of meaningful amounts of any of these cytokines (Figure 7A-D, white bars). In contrast, all these cytokines were produced abundantly by DC treated with SOSIP.R6-IZ-CD40L (763, 375, 191 and 523 pg/ml, respectively) or IZ-CD40L (675, 123, 50 and 215 pg/ml, respectively). As expected, the TNF-α/IL-1β/LPS maturation cocktail also induced IL-6, IL-10 and IL-12 secretion, albeit with some qualitative differences compared to SOSIP.R6-IZ-CD40L. TNF-α induction could not be analyzed because it was already present in the maturation cocktail. DC stimulated with TNF-α/IL-1β secreted moderate amounts of IL-6 and low levels of IL-10 and IL-12, and the addition of mock or SOSIP.R6-IZ supernatant had no further effect (Figure 7A-D, black bars). However, the addition of SOSIP.R6-IZ-CD40L or IZ-CD40L to the TNF-α/IL-1β cocktail substantially increased the secretion of all three cytokines.

### SOSIP.R6-IZ-CD40L-exposed DC prime naïve CD4^+ ^T-cells

**Figure 8:**
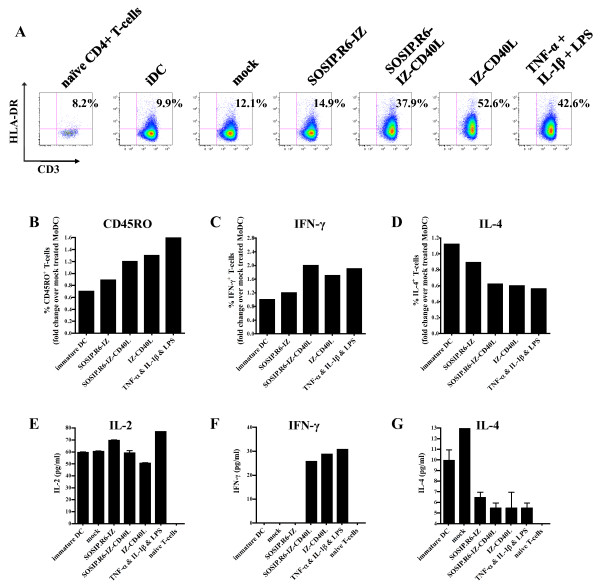
**SOSIP.R6-IZ-CD40L-exposed DC prime naïve CD4^+ ^T-cells**. **A**. HLA-DR expression on CD4+ T-cells cultured for 5 days in the absence or presence of DC that were exposed to SOSIP.R6-IZ, SOSIP.R6-IZ-CD40L, or control stimuli was measured by FACS. Percentages of CD3^+ ^HLA-DR^+ ^T-cells are displayed within each graph. Relative number of T cells positive for cell surface expression of CD45RO (the percentages of positive cells are given in the text) **(B)**, and intracellular expression of IFN-γ **(C) **and IL-4 **(D)**. IL-2 **(E)**, IFN-γ **(F) **and IL-4 **(G) **levels in the supernatant of DC-T cell co-cultures at day 5 measured by ELISA. Data are representative for three independent experiments.

After stimulation with the various stimuli outlined above, DC were co-cultured for 5 days with allogeneic naïve CD4^+ ^T cells in a mixed lymphocyte reaction to investigate their T_H_-priming capacity. Expression of the late activation marker HLA-DR (MHC class II) on the CD4^+ ^T cells was then analyzed (Figure 8A). DCs exposed to the mock supernatant or SOSIP.R6-IZ stimulated CD4^+ ^T-cells only poorly (8.6% and 11.4% HLA-DR^+ ^T cells, respectively, compared to 2.6% for unstimulated T cells and 4.5% for T cells co-cultured with iDC). In contrast, DC that had been matured with SOSIP.R6-IZ-CD40L or IZ-CD40L induced HLA-DR upregulation on 31.6% and 46.7% of the CD4^+ ^T cells, respectively. This degree of HLA-DR up-regulation was higher than on T cells co-cultured with TNF-α/IL-1β/LPS-matured DC (24.4% HLA-DR^+^). We, next, investigated whether the T cells expressed the memory T cell marker CD45RO (Figure 8B). 11% of the CD4^+ ^T cells that were co-cultured with SOSIP.R6-IZ-treated DC expressed CD45RO, while 19% CD4^+ ^T cells that were co-cultured with TNF-α/IL-1β/LPS-matured DC stained positive for CD45RO and 9% of the CD4^+ ^T cells incubated with iDC. The SOSIP.R6-IZ-CD40L-exposed DC induced a subtly enhanced expression of CD45RO (14% positive CD4^+ ^T cells), similar to IZ-CD40L-exposed DC (16%). Thus, compared to SOSIP.R6-IZ-treated DC, SOSIP.R6-IZ-CD40L-exposed DC induced enhanced numbers of CD4^+ ^T cells positive for the activation marker HLA-DR and the memory marker CD45RO.

To assess the CD4^+ ^T cell polarization, we stained the CD4^+ ^T cells for intracellular IFN-γ and IL-4 (Figure 8C, D). We noted that SOSIP.R6-IZ-CD40L-exposed DC induced twice the number IFN-γ expressing CD4^+ ^T cells compared to SOSIP.R6-IZ- or mock-treated DC, similar to the numbers induced by TNF-α/IL-1β/LPS- and IZ-CD40L-matured DC (Figure 8C). The enhanced expression of IFN-γ was mirrored by a decreased expression of IL-4 (Figure 8D). The intracellular staining data were corroborated by ELISA experiments. Unstimulated CD4^+ ^T cells or cells co-cultured with iDC, mock-treated DC or SOSIP.R6-IZ-treated DC did not secrete detectable levels of IFN-γ (Figure 8F). However, DC exposed to SOSIP.R6-IZ-CD40L or IZ-CD40L induced the release of significant amounts of IFN-γ from CD4^+ ^T-cells (26 and 29 pg/ml, respectively), as did TNF-α/IL-1β/LPS-matured DC (31 pg/ml). Consistent with the intracellular staining data, the increase in IFN-γ was associated with a decrease in IL-4 secretion (Figure 8G). We also measured IL-2 secretion in DC-T cell co-cultures and found that SOSIP.R6-IZ-CD40L-treated DC behaved no differently from cells exposed to SOSIP.R6-IZ (Figure 8E). In summary, DC exposed to SOSIP.R6-IZ-CD40L can prime naïve CD4^+ ^T-cells in vitro and induce them to secrete IFN-γ.

## Discussion

To counter the poor immunogenicity of HIV-1 Env proteins, we fused stabilized SOSIP.R6 trimers directly to a costimulatory molecule, CD40L, the latter acting as a '*cis*-adjuvant' to activate DC. We show here that the chimeric SOSIP.R6-IZ-CD40L molecule is folded correctly, can engage CD40 and signal through CD40 to activate human DC. In turn, SOSIP.R6-IZ-CD40L-exposed DC are able to activate CD4^+ ^T-cells, and induce a pool of memory T cells. These data suggest that SOSIP.R6-IZ-CD40L may induce enhanced immune responses including memory responses and warrant the *in vivo *testing of this construct.

We intend to use SOSIP.R6-IZ-CD40L trimers for subsequent immunogenicity studies. The *cis*-adjuvant strategy described here differs from approaches using lectin receptors to target DC and enhance antigen presentation [24-27]. First, CD40L not only targets the antigen to DC, but also activates these cells. Second, CD40L also activates cell types other than DC, notably B cells. The covalent linkage of an adjuvant and antigen has been shown to be a more effective way to enhance antigen-specific immune responses than a simple mixture of adjuvant and antigen. For example, immunization with HIV Gag, chemically linked to a TLR7/8 agonist, induced substantially better humoral and cellular responses than Gag that was merely mixed with the same adjuvant [19]. As another example, a GFP-CD40L fusion protein was superior to GFP or CD40L alone and the GFP + CD40L mixture for inducing GFP-specific antibody responses in mice [60]. Similarly, CD40L fusion enhanced the immunogenicity of a self (tumor) antigen in mice, bovine herpesvirus-1 glycoprotein D in cattle, and duck hepatitis B virus core antigen in ducks [61-64]. The likely explanation for the benefit incurred by covalently linking antigen to the adjuvant is that the very cells encountering the antigen are simultaneously activated by the adjuvant.

Incorporation of CD40L in canarypox vectors, expressing HIV antigens, has also been shown to benefit immune responses [43]. Furthermore, recent studies have shown that CD40L can be effectively incorporated into HIV virus-like particles (VLPs) [41,42]. Such VLPs were shown to stimulate DC and were able to induce improved humoral and cellular responses in mice compared to VLPs that did not contain CD40L.

One concern with using host molecules to enhance vaccine immunogenicity is the potential induction of auto-antibodies. When CD40L was tested in a phase I trial to treat cancer patients, no major side effects were observed [44]. We also note that the amounts of protein required for vaccination are at least 2 orders of magnitude lower than those required in a therapeutic setting, which should reduce the chances of inducing auto-antibodies. 3 injections of 20-150 μg of a typical protein vaccine confer protection against infection. In phase III trials using gp120, an unusual amount and number of doses was given (7 injections of 600 μg gp120), but this is still considerably lower than the amounts used as therapeutics. In the above mentioned clinical trial using CD40L, patients received 50-150 μg of CD40L per kg bodyweight per day. Nevertheless, when evaluating the CD40L fusion construct in preclinical in vivo studies and potential clinical trials, the induction of antibodies against CD40L should be closely monitored.

A good humoral response to an antigen should involve antibodies of high titer, affinity and avidity, and should have a long half-life with the creation of B cell memory (and preferably also T cell memory). These various outcomes may be facilitated by the use of CD40L and/or other *cis*-adjuvants. Stimulation of the immune system by adjuvants or '*cis*-adjuvants' increases the overall level of antibodies to the administered antigen, but only a subset of these antibodies is neutralizing. This can be beneficial, by analogy to the aphorism that 'a rising tide lifts all boats'. However, providing additional stimulatory signals to B cells may also improve the quality of the antibodies that are elicited, perhaps because B cell stimulation helps the affinity maturation process [18]. Thus, a recent study on RSV demonstrates that B cell stimulation through TLRs improves both the affinity and neutralizing potency of anti-RSV antibodies [18].

## Conclusion

In conclusion, we have characterized a trimeric gp140-CD40L fusion protein that is able to activate human DC whereas unconjugated gp140 is not able to do so. Overall, the use of '*cis*-adjuvants' may have wider applicability in subunit vaccine development. The wealth of natural candidate molecules and the increasing understanding of their roles in immunology provide an opportunity for customized immunogen design in which the antigen is coupled to a molecule to enhance a specific and desired type of response.

## Competing interests

The authors declare that they have no competing interests.

## Authors' contributions

Author contributions are as follows. MM contributed to study design and performed experiments. KM, RDV, DE, TVM, IB, CVDS, KD performed experiments. BB, JPM and RWS conceived and designed the study. MM, BB, JPM and RWS wrote the manuscript. All authors read and approved the final manuscript.

## References

[B1] Reynolds MR Weiler AM Weisgrau KL Piaskowski SM Furlott JR Weinfurter JT Kaizu M Soma T Leon EJ MacNair C Leaman DP Zwick MB Gostick E Musani SK Price DA Friedrich TC Rakasz EG Wilson NA McDermott AB Boyle R Allison DB Burton DR Koff WC Watkins DI Macaques vaccinated with live-attenuated SIV control replication of heterologous virus J Exp Med 2008 205 2537 2550 10.1084/jem.20081524 18838548 PMC2571929

[B2] Liu J O'Brien KL Lynch DM Simmons NL La PA Riggs AM Abbink P Coffey RT Grandpre LE Seaman MS Landucci G Forthal DN Montefiori DC Carville A Mansfield KG Havenga MJ Pau MG Goudsmit J Barouch DH Immune control of an SIV challenge by a T-cell-based vaccine in rhesus monkeys Nature 2009 457 87 91 10.1038/nature07469 18997770 PMC2614452

[B3] Shattock RJ Haynes BF Pulendran B Flores J Esparza J Improving defences at the portal of HIV entry: mucosal and innate immunity PLoS Med 2008 5 e81 10.1371/journal.pmed.0050081 18384232 PMC2276525

[B4] Flynn NM Forthal DN Harro CD Judson FN Mayer KH Para MF Placebo-controlled phase 3 trial of a recombinant glycoprotein 120 vaccine to prevent HIV-1 infection J Infect Dis 2005 191 654 665 15688278 10.1086/428404

[B5] Pitisuttithum P Gilbert P Gurwith M Heyward W Martin M van GF Hu D Tappero JW Choopanya K Randomized, double-blind, placebo-controlled efficacy trial of a bivalent recombinant glycoprotein 120 HIV-1 vaccine among injection drug users in Bangkok, Thailand J Infect Dis 2006 194 1661 1671 10.1086/508748 17109337

[B6] Burton DR Desrosiers RC Doms RW Koff WC Kwong PD Moore JP Nabel GJ Sodroski J Wilson IA Wyatt RT HIV vaccine design and the neutralizing antibody problem Nat Immunol 2004 5 233 236 10.1038/ni0304-233 14985706

[B7] Eggink D Melchers M Sanders RW Antibodies to HIV-1: aiming at the right target Trends Microbiol 2007 15 291 294 10.1016/j.tim.2007.05.006 17566740

[B8] Kwong PD Wilson IA HIV-1 and influenza antibodies: seeing antigens in new ways Nat Immunol 2009 10 573 578 10.1038/ni.1746 19448659 PMC2796958

[B9] Parren PW Burton DR Sattentau QJ HIV-1 antibody--debris or virion? Nat Med 1997 3 366 367 9095159 10.1038/nm0497-366d

[B10] Walker LM Burton DR Rational antibody-based HIV-1 vaccine design: current approaches and future directions Curr Opin Immunol 2010 22 3 358 66 Epub 2010 Mar 17 10.1016/j.coi.2010.02.012 20299194 PMC2891291

[B11] Binley JM Sanders RW Clas B Schuelke N Master A Guo Y Kajumo F Anselma DJ Maddon PJ Olson WC Moore JP A recombinant HIV-1 envelope glycoprotein complex stabilized by an intermolecular disulfide bond between the gp120 and gp41 subunits is an antigenic mimic of the trimeric virion-associated structure J Virol 2000 74 627 643 10.1128/JVI.74.2.627-643.2000 10623724 PMC111582

[B12] Sanders RW Vesanen M Schuelke N Master A Schiffner L Kalyanaraman R Paluch M Berkhout B Maddon PJ Olson WC Lu M Moore JP Stabilization of the soluble, cleaved, trimeric form of the envelope glycoprotein complex of human immunodeficiency virus type 1 J Virol 2002 76 8875 8889 10.1128/JVI.76.17.8875-8889.2002 12163607 PMC136973

[B13] Yang X Wyatt R Sodroski J Improved elicitation of neutralizing antibodies against primary human immunodeficiency viruses by soluble stabilized envelope glycoprotein trimers J Virol 2001 75 1165 1171 10.1128/JVI.75.3.1165-1171.2001 11152489 PMC114022

[B14] Srivastava IK Stamatatos L Kan E Vajdy M Lian Y Hilt S Martin L Vita C Zhu P Roux KH Vojtech L C Montefiori D Donnelly J Ulmer JB Barnett SW Purification, characterization, and immunogenicity of a soluble trimeric envelope protein containing a partial deletion of the V2 loop derived from SF162, an R5-tropic human immunodeficiency virus type 1 isolate J Virol 2003 77 11244 11259 10.1128/JVI.77.20.11244-11259.2003 14512572 PMC224963

[B15] Beddows S Franti M Dey AK Kirschner M Iyer SP Fisch DC Ketas T Yuste E Desrosiers RC Klasse PJ Maddon PJ Olson WC Moore JP A comparative immunogenicity study in rabbits of disulfide-stabilized, proteolytically cleaved, soluble trimeric human immunodeficiency virus type 1 gp140, trimeric cleavage-defective gp140 and monomeric gp120 Virol 2007 360 329 340 10.1016/j.virol.2006.10.032 17126869

[B16] Gilbert PB Peterson ML Follmann D Hudgens MG Francis DP Gurwith M Heyward WL Jobes DV Popovic V Self SG Sinangil F Burke D Berman PW Correlation between immunologic responses to a recombinant glycoprotein 120 vaccine and incidence of HIV-1 infection in aphase 3 HIV-1 preventive vaccine trial J Infect Dis 2005 191 666 677 10.1086/428405 15688279

[B17] Bonsignori M Moody MA Parks RJ Holl TM Kelsoe G Hicks CB Vandergrift N Tomaras GD Haynes BF HIV-1 envelope induces memory B cell responses that correlate with plasma antibody levels after envelope gp120 protein vaccination or HIV-1 infection J Immunol 2009 183 2708 2717 10.4049/jimmunol.0901068 19625640 PMC3089979

[B18] Delgado MF Coviello S Monsalvo AC Melendi GA Hernandez JZ Batalle JP Diaz L Trento A Chang HY Mitzner W Ravetch J Melero JA Irusta PM Polack FP Lack of antibody affinity maturation due to poor Toll-like receptor stimulation leads to enhanced respiratory syncytial virus disease Nat Med 2009 15 34 41 10.1038/nm.1894 19079256 PMC2987729

[B19] Wille-Reece U Flynn BJ Lore K Koup RA Kedl RM Mattapallil JJ Weiss WR Roederer M Seder RA HIV Gag protein conjugated to a Toll-like receptor 7/8 agonist improves the magnitude and quality of Th1 and CD8+ T cell responses in nonhuman primates Proc Natl Acad Sci USA 2005 102 15190 15194 10.1073/pnas.0507484102 16219698 PMC1257732

[B20] Eckl-Dorna J Batista FD BCR-mediated uptake of antigen linked to TLR9 ligand stimulates B-cell proliferation and antigen-specific plasma cell formation Blood 2009 113 3969 3977 10.1182/blood-2008-10-185421 19144984

[B21] Bower JF Yang X Sodroski J Ross TM Elicitation of neutralizing antibodies with DNA vaccines expressing soluble stabilized human immunodeficiency virus type 1 envelope glycoprotein trimers conjugated to C3d J Virol 2004 78 4710 4719 10.1128/JVI.78.9.4710-4719.2004 15078953 PMC387675

[B22] Koch M Frazier J Sodroski J Wyatt R Characterization of antibody responses to purified HIV-1 gp120 glycoproteins fused with the molecular adjuvant C3d Virol 2005 340 277 284 10.1016/j.virol.2005.06.034 16051303

[B23] McCormick AL Thomas MS Heath AW Immunization with an interferon-gamma-gp120 fusion protein induces enhanced immune responses to human immunodeficiency virus gp120 J Inf Dis 2001 184 1423 1430 10.1086/324371 11709784

[B24] Bonifaz LC Bonnyay DP Charalambous A Darguste DI Fujii S Soares H Brimnes MK Moltedo B Moran TM Steinman RM In vivo targeting of antigens to maturing dendritic cells via the DEC-205 receptor improves T cell vaccination J Exp Med 2004 199 815 824 10.1084/jem.20032220 15024047 PMC2212731

[B25] Dudziak D Kamphorst AO Heidkamp GF Buchholz VR Trumpfheller C Yamazaki S Cheong C Liu K Lee HW Park CG Steinman RM Nussenzweig MC Differential antigen processing by dendritic cell subsets in vivo Science 2007 315 107 111 10.1126/science.1136080 17204652

[B26] Yang L Yang H Rideout K Cho T Joo KI Ziegler L Elliot A Walls A Yu D Baltimore D Wang P Engineered lentivector targeting of dendritic cells for in vivo immunization Nat Biotechnol 2008 26 326 334 10.1038/nbt1390 18297056 PMC2366162

[B27] Lahoud MH Proietto AI Ahmet F Kitsoulis S Eidsmo L Wu L Sathe P Pietersz S Chang HW Walker ID Maraskovsky E Braley H Lew AM Wright MD Heath WR Shortman K Caminschi I The C-type lectin Clec12A present on mouse and human dendritic cells can serve as a target for antigen delivery and enhancement of antibody responses J Immunol 2009 182 7587 7594 10.4049/jimmunol.0900464 19494282

[B28] Ma DY Clark EA The role of CD40 and CD154/CD40L in dendritic cells Semin Immunol 2009 21 265 272 10.1016/j.smim.2009.05.010 19524453 PMC2749083

[B29] Caux C Massacrier C Vanbervliet B Dubois B van KC Durand I Banchereau J Activation of human dendritic cells through CD40 cross-linking J Exp Med 1994 180 1263 1272 10.1084/jem.180.4.1263 7523569 PMC2191669

[B30] S hreedhar V Moodycliffe AM Ullrich SE Bucana C Kripke ML Flores-Romo L Dendritic cells require T cells for functional maturation in vivo Immunity 1999 11 625 636 10.1016/S1074-7613(00)80137-5 10591187

[B31] van Kooten C Banchereau J CD40-CD40 ligand J Leukoc Biol 2000 67 2 17 10647992 10.1002/jlb.67.1.2

[B32] Gray D Siepmann K Wohlleben G CD40 ligation in B cell activation, isotype switching and memory development Semin Immunol 1994 6 303 310 10.1006/smim.1994.1039 7532460

[B33] Mendoza RB Cantwell MJ Kipps TJ Immunostimulatory effects of a plasmid expressing CD40 ligand (CD154) on gene immunization J Immunol 1997 159 5777 5781 9550372

[B34] Dullforce P Sutton DC Heath AW Enhancement of T cell-independent immune responses in vivo by CD40 antibodies Nat Med 1998 4 88 91 10.1038/nm0198-088 9427612

[B35] Gurunathan S Irvine KR Wu CY Cohen JI Thomas E Prussin C Restifo NP Seder RA CD40 ligand/trimer DNA enhances both humoral and cellular immune responses and induces protective immunity to infectious and tumor challenge J Immunol 1998 161 4563 4571 9794383 PMC2239005

[B36] Rolph MS Kaufmann SH CD40 signaling converts a minimally immunogenic antigen into a potent vaccine against the intracellular pathogen Listeria monocytogenes J Immunol 2001 166 5115 5121 11290793 10.4049/jimmunol.166.8.5115

[B37] Ninomiya A Ogasawara K Kajino K Takada A Kida H Intranasal administration of a synthetic peptide vaccine encapsulated in liposome together with an anti-CD40 antibody induces protective immunity against influenza A virus in mice Vaccine 2002 20 3123 3129 10.1016/S0264-410X(02)00261-X 12163263

[B38] Staveley-O'Carroll K Schell TD Jimenez M Mylin LM Tevethia MJ Schoenberger SP Tevethia SS In vivo ligation of CD40 enhances priming against the endogenous tumor antigen and promotes CD8+ T cell effector function in SV40 T antigen transgenic mice J Immunol 2003 171 697 707 12847236 10.4049/jimmunol.171.2.697

[B39] Wienhold D Armengol E Marquardt A Marquardt C Voigt H Buttner M Saalmuller A Pfaff E Immunomodulatory effect of plasmids co-expressing cytokines in classical swine fever virus subunit gp55/E2-DNA vaccination Vet Res 2005 36 571 587 10.1051/vetres:2005019 15955282

[B40] Llopiz D Dotor J Zabaleta A Lasarte JJ Prieto J Borras-Cuesta F Sarobe P Combined immunization with adjuvant molecules poly(I:C) and anti-CD40 plus a tumor antigen has potent prophylactic and therapeutic antitumor effects Cancer Immunol Immunother 2008 57 19 29 10.1007/s00262-007-0346-8 17564702 PMC11029881

[B41] Zhang R Zhang S Li M Chen C Yao Q Incorporation of CD40 ligand into SHIV virus-like particles (VLP) enhances SHIV-VLP-induced dendritic cell activation and boosts immune responses against HIV Vaccine 2010 28 31 5114 27 Epub 2010 May 14 10.1016/j.vaccine.2010.03.079 20471443 PMC2906648

[B42] Skountzou I Quan FS Gangadhara S Ye L Vzorov A Selvaraj P Jacob J Compans RW Kang SM Incorporation of glycosylphosphatidylinositol-anchored granulocyte-macrophage colonystimulating factor or CD40 ligand enhances immunogenicity of chimeric simian immunodeficiency virus-like particles J Virol 2007 81 1083 1094 10.1128/JVI.01692-06 17108046 PMC1797543

[B43] Liu J Yu Q Stone GW Yue FY Ngai N Jones RB Kornbluth RS Ostrowski MA CD40L expressed from the canarypox vector, ALVAC, can boost immunogenicity of HIV-1 canarypox vaccine in mice and enhance the in vitro expansion of viral specific CD8+ T cell memory responses from HIV-1-infected and HIV-1-uninfected individuals Vaccine 2008 26 4062 4072 10.1016/j.vaccine.2008.05.018 18562053 PMC3060027

[B44] Vonderheide RH Dutcher JP Anderson JE Eckhardt SG Stephans KF Razvillas B Garl S Butine MD Perry VP Ghalie R Caron DA Gribben JG Phase I study of recombinant human CD40 ligand in cancer patients Journal of Clinical Oncology 2001 19 3280 3287 11432896 10.1200/JCO.2001.19.13.3280

[B45] Binley JM Sanders RW Master A Cayanan CS Wiley CL Schiffner L Travis B Kuhmann S Burton DR Hu S-L Olsos WC Moore JP Enhancing the proteolytic maturation of human immunodeficiency virus type 1 envelope glycoproteins J Virol 2002 76 2606 2616 10.1128/JVI.76.6.2606-2616.2002 11861826 PMC135977

[B46] Harbury PB Kim PS Alber T Crystal structure of an isoleucine-zipper trimer Nature 1994 371 80 83 10.1038/371080a0 8072533

[B47] Eggink D Melchers M Wuhrer M van MT Dey AK Naaijkens BA David KB Le D Deelder AM Kang K Olson WC Berkhout B Hokke CH Moore JP Sanders RW Lack of complex N-glycans on HIV-1 envelope glycoproteins preserves protein conformation and entry function Virol 2010 401 236 247 10.1016/j.virol.2010.02.019 PMC3776475 20304457

[B48] Kirschner M Monrose V Paluch M Techodamrongsin N Rethwilm A Moore JP The production of cleaved, trimeric human immunodeficiency virus type 1 (HIV-1) envelope glycoprotein vaccine antigens and infectious pseudoviruses using linear polyethylenimine as a transfection reagent Protein Expr Purif 2006 48 61 68 10.1016/j.pep.2006.02.017 16600625

[B49] Trkola A Dragic T Arthos J Binley JM Olson WC Allaway GP Cheng-Mayer C Robinson J Maddon PJ Moore JP CD4-dependent, antibody-sensitive interactions between HIV-1 and its co-receptor CCR-5 Nature 1996 384 184 187 10.1038/384184a0 8906796

[B50] Schagger H Cramer WA von JG Analysis of molecular masses and oligomeric states of protein complexes by blue native electrophoresis and isolation of membrane protein complexes by two-dimensional native electrophoresis Anal Biochem 1994 217 220 230 10.1006/abio.1994.1112 8203750

[B51] Schagger H von JG Blue native electrophoresis for isolation of membrane protein complexes in enzymatically active form Anal Biochem 1991 199 223 231 10.1016/0003-2697(91)90094-A 1812789

[B52] Yang X Florin L Farzan M Kolchinsky P Kwong PD Sodroski J Wyatt R Modifications that stabilize human immunodeficiency virus envelope glycoprotein trimers in solution J Virol 2000 74 4746 4754 10.1128/JVI.74.10.4746-4754.2000 10775613 PMC111997

[B53] Pancera M Lebowitz J Schon A Zhu P Freire E Kwong PD Roux KH Sodroski J Wyatt R Soluble mimetics of human immunodeficiency virus type 1 viral spikes produced by replacement of the native trimerization domain with a heterologous trimerization motif: characterization and ligand binding analysis J Virol 2005 79 9954 9969 10.1128/JVI.79.15.9954-9969.2005 16014956 PMC1181572

[B54] Si Z Phan N Kiprilov E Sodroski J Effects of HIV type 1 envelope glycoprotein proteolytic processing on antigenicity AIDS Res Hum Retroviruses 2003 19 217 226 10.1089/088922203763315722 12689414

[B55] Morris AE Remmele RL Klinke R Macduff BM Fanslow WC Armitage RJ Incorporation of an isoleucine zipper motif enhances the biological activity of soluble CD40L (CD154) J Biol Chem 1999 274 418 423 10.1074/jbc.274.1.418 9867859

[B56] Stone GW Barzee S Snarsky V Spina CA Lifson JD Pillai VK Amara RR Villinger F Kornbluth RS Macaque multimeric soluble CD40 ligand and GITR ligand constructs are immunostimulatory molecules in vitro Clin Vaccine Immunol 2006 13 1223 1230 10.1128/CVI.00198-06 16988005 PMC1656546

[B57] Haswell LE Glennie MJ Al-Shamkhani A Analysis of the oligomeric requirement for signaling by CD40 using soluble multimeric forms of its ligand, CD154 Eur J Immunol 2001 31 3094 3100 10.1002/1521-4141(2001010)31:10<3094::AID-IMMU3094>3.0.CO;2-F 11592086

[B58] Maurais E Cantin R Tremblay MJ Human immunodeficiency virus type 1-anchored CD40 ligand induces secretion of the chemokine interleukin-8 by human primary macrophages Virol 2009 385 227 232 10.1016/j.virol.2008.11.033 19101003

[B59] Shan M Klasse PJ Banerjee K Dey AK Iyer SP Dionisio R Charles D Campbell-Gardener L Olson WC Sanders RW Moore JP HIV-1 gp120 mannoses induce immunosuppressive responses from dendritic cells PLoS Pathog 2007 3 e169 10.1371/journal.ppat.0030169 17983270 PMC2048530

[B60] Li W Synergistic antibody induction by antigen-CD40 ligand fusion protein as improved immunogen Immunology 2005 115 215 222 10.1111/j.1365-2567.2005.02141.x 15885127 PMC1782153

[B61] Chen HW Huang HI Lee YP Chen LL Liu HK Cheng ML Tsai JP Tao MH Ting CC Linkage of CD40L to a self-tumor antigen enhances the antitumor immune responses of dendritic cellbased treatment Cancer Immunol Immunother 2002 51 341 348 10.1007/s00262-002-0283-5 12111122 PMC11032834

[B62] Huang HI Wu PY Teo CY Chen MN Chen YC Silin D Tao MH Improved immunogenicity of a self tumor antigen by covalent linkage to CD40 ligand International Journal of Cancer 2004 108 696 703 10.1002/ijc.11612 14696096

[B63] Manoj S Griebel PJ Babiuk LA Littel-Van Den Hurk SV Modulation of immune responses to bovine herpesvirus-1 in cattle by immunization with a DNA vaccine encoding glycoprotein D as a fusion protein with bovine CD154 Immunology 2004 112 328 338 10.1111/j.1365-2567.2004.01877.x 15147576 PMC1782479

[B64] Gares SL Fischer KP Congly SE Lacoste S Addison WR Tyrrell DL Gutfreund KS Immunotargeting with CD154 (CD40 ligand) enhances DNA vaccine responses in ducks Clin Vaccine Immunol 2006 13 958 965 10.1128/CVI.00080-06 16893998 PMC1539120

